# Operative challenges in a gigantic ganglioneuroma of the posterior mediastinum with mediastinal compression

**DOI:** 10.1093/jscr/rjad524

**Published:** 2023-09-20

**Authors:** Muhammad Abid Amir, Mohamed Izzad Isahak, Isqandar Adnan, Mohd Zamrin Dimon

**Affiliations:** Department of Cardiovascular and Thoracic Surgery, Faculty of Medicine, Universiti Teknologi MARA Sungai Buloh Campus, 47000 Selangor, Malaysia; Department of Cardiovascular and Thoracic Surgery, Faculty of Medicine, Universiti Teknologi MARA Sungai Buloh Campus, 47000 Selangor, Malaysia; Department of Anaesthesiology, Faculty of Medicine, Universiti Teknologi MARA Sungai Buloh Campus, 47000 Selangor, Malaysia; Department of Cardiovascular and Thoracic Surgery, Faculty of Medicine, Universiti Teknologi MARA Sungai Buloh Campus, 47000 Selangor, Malaysia

## Abstract

Symptomatic giant ganglioneuromas with mediastinal compression are rare, complicating its management with significant morbidity and mortality risks. A meticulous multidisciplinary preoperative planning is pivotal in ensuring success. We describe a case of a 30-year-old man with a giant posterior mediastinal mass with compression and displacement of the mediastinal structures. Biopsy confirmed a ganglioneuroma and patient underwent excision. Surgery was challenging in view of the size and adherence to the local structures. Haemodynamic instabilities were encountered necessitating a pre-emptive femoral-femoral cannulation for CPB. A piece-meal debulking of the tumour was performed, complicated with massive haemorrhage requiring autologous blood transfusion using an intraoperative blood salvage device. The patient recovered and was discharged home well at Day 8. A thorough pre-operative planning involving a multidisciplinary approach, an understanding of the surgical anatomy as well as anticipating impending complications is of paramount importance  in the management of this particular case.

## Introduction

Tumours of the posterior mediastinum are commonly neurogenic in origin. These can arise from the cells of the nerve sheath, paraganglionic tissue and autonomic ganglia, all of which originate embryonically from the neural crest. Ganglioneuromas are rare benign tumours arising from the sympathetic ganglions [1]. About 40% of these tumours are found in the posterior mediastinum. Other locations that it has been reported include the tongue, bladder, uterus and skin. They are typically asymptomatic and are usually discovered incidentally. However, symptoms are dependent on its location and size as well as the ability to secrete hormones. Large tumours of the mediastinum can result in dypsnoea, chest pain as well as spinal cord compression leading to weakness and pain of the extremities. The production of catecholamines may result in hypertension and flushing.

## Case presentation

A 30-year-old Burmese gentleman presented with reduced effort tolerance of 4 months duration worse on exertion. It was associated with positional dyspnea, mainly on the right lateral position as well as intermittent non-productive cough of similar duration. There was an absence of fever, hemoptysis and weight loss. He was a non-smoker with no other significant medical, surgical and family history to date. His physical examination was unremarkable except for reduced air entry over the upper half of the right hemithorax. Upon presentation, a chest radiograph done showed a large soft tissue mass occupying the right hemithorax mainly over the upper and middle zones. His laboratory tests were otherwise within normal limits.

**Figure 1:**
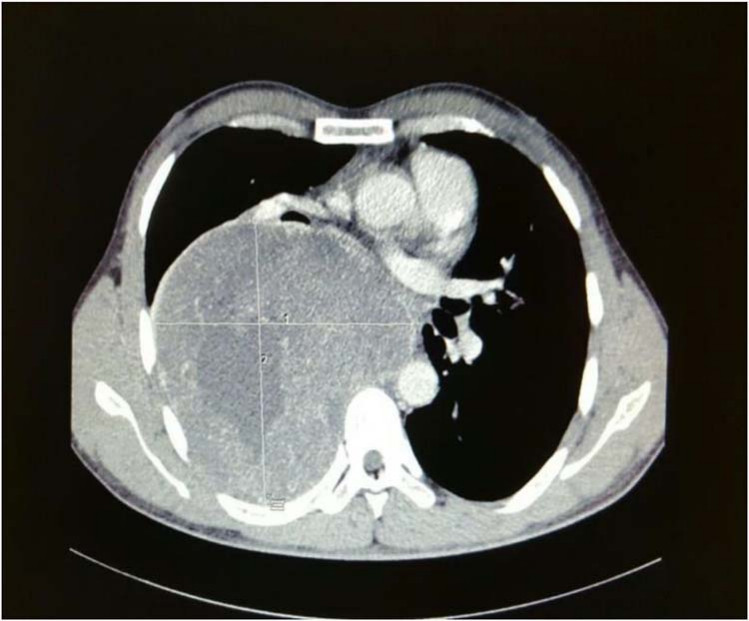
Axial cut of a contrasted CT Thorax depicting a large right posterior mediastinal mass.

Computed tomography of the chest depicted a large well-defined heterogeneously enhancing right posterior mediastinal mass which measured 13.1 cm (width) × 12.6 cm (antero-posterior) × 17 cm (cranio-caudal), with foci of calcification, increased vascularity and areas of necrosis (Fig. 1).

The right main pulmonary artery and right main bronchus were splayed and narrowed, with the esophagus displaced to the left. It extended superiorly to the mid-tracheal level. Although there was no evidence of right sided neuroforaminal widening, there was pressure erosion of right T4 and T5 posterior ribs. A bronchoscopy performed revealed external compression affecting the right and left main bronchus and part of the carina, with no evidence of bronchomalacia. A baseline lung function test done also showed severe obstructive pattern with an FEV1 of 1.11 L (29% predicted), an FVC of 2.29 L (50% predicted) as well as an FEV1/FVC ratio of 49. A CT-guided biopsy of the posterior mediastinal mass was subsequently performed, and histopathological assessment showed presence of spindled-shaped and ganglion-like cells with no evidence of malignancy, suggestive of ganglioneuroma.

In view of the histopathological findings as well as progressively worsening symptoms, a decision was made for surgery. A multidisciplinary meeting and discussions were held preoperatively highlighting the possible complications encountered, mainly in view of the massive size and proximity to the surrounding mediastinal structures. Preoperative concerns were addressed among the multidisciplinary teams (MDT) involved, including difficult airway management, cardiorespiratory collapse upon induction and the need for CPB support, risks of trachea/bronchomalacia and major hemorrhage, necessitating a preoperative angioembolization of the tumor.

**Figure 2:**
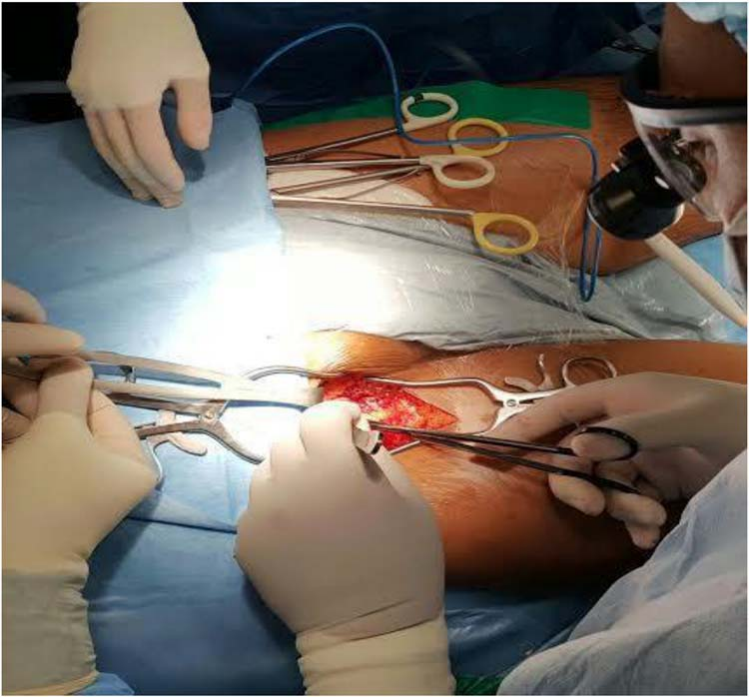
Right femoral-femoral access for cardiopulmonary bypass.

The patient was then taken to the operating room, and a right posterolateral thoracotomy was performed. A huge mass was seen occupying the whole right hemithorax, necessitating removal of the fifth rib to facilitate exposure. The mass was immobile, adherent mainly posteriorly to the paravertebral region. The right lung, pulmonary hilum and other vital mediastinal structures were free and not invaded by the large tumor. Surgery was difficult in view of the size, vascularity as well as its adherence, posteriorly. Frequent episodes of hemodynamic instability were encountered during handling and mobilisation, necessitating a pre-emptive fem-fem cannulation for CPB (Fig. 2).

**Figure 3:**
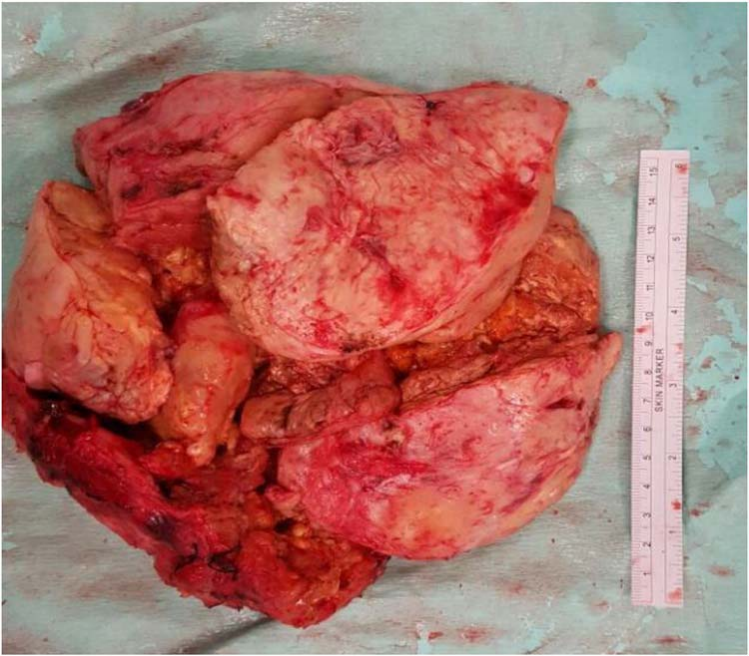
A photo micrograph of the surgical specimen removed.

A piece-meal debulking of the tumor was subsequently done, complicated with massive hemorrhage requiring autologous blood transfusion using an intraoperative blood salvage device. Gross examination of the specimen revealed a yellowish tan firm tumor mass in multiple pieces measuring 21 cm × 22 cm × 6 cm in aggregate diameter (Fig. 3). The outer surface is smooth with a grayish pale appearance, while the cut section has a yellowish fleshy surface.

Histologically, the tumor comprises of spindle cell (Schwannian) proliferation with the presence of ganglion cells in clusters and multinucleated form. No evidence of malignancy was seen. These findings led to the final diagnosis of a posterior mediastinal ganglioneuroma.

Although the patient had an eventful post-operative recovery mainly due to the complication of massive blood transfusion, he was well enough to be discharged home at day 8 post operatively.

## Discussion

Ganglioneuroma is a rare tumor, originating from the neural crest cells. It mainly arises from the autonomic ganglion cells of the peripheral nervous system, commonly the sympathetic ganglion. It is a benign tumor whereas a ganglioneuroblastoma represents the malignant end of the ganglion cell lineage. This category of neoplasms also includes peripheral nerve sheath tumors, melanomas and neuroendocrine cell tumors [2]. Ganglioneuromas can arise anywhere along the peripheral autonomic ganglion sites and is most seen in the posterior mediastinum and retroperitoneum area. Other rare areas include the adrenal medulla, tongue, bladder, uterus, bone and skin [3]. It can also secrete active hormones such as vasoactive intestinal polypeptide, catecholamines and testosterone [4]. It is often seen in children and adolescents with no difference in distribution among males and females [5].

Ganglioneuromas are commonly asymptomatic and seen incidentally on chest imaging. It grows slowly until it reaches a bigger size, resulting in compressive symptoms or even invasion into the surrounding mediastinal structures and spine. These rare presentations may result in symptoms such as chest pain due to compression on the thoracic bone, reduced effort tolerance, dyspnea, cough or hemoptysis. It can also secrete hormones such as catecholamines resulting in hypertension and flushing [6]. In our patient, his symptoms were predominantly due to the mass effect and compression over the lung and airway resulting in exertional and positional dyspnea.

The management of ganglioneuromas is complete surgical resection to achieve a complete cure [7]. Local recurrence is uncommon. This can be done safely and will normally result in an excellent prognosis [8]. However, in our case, we would like to highlight the unusually gigantic size of our tumor with compression of vital mediastinal structures as well as more than one tumor pedicle attached to the vertebral bodies. This necessitated a multidisciplinary approach involving the cardiothoracic surgeons, thoracic radiologists, spine surgeons, respiratory physicians, anesthetist as well as perfusionists. The preparation for airway stenting in the event of tracheobonchomalacia, preoperative angio-embolization, pre-emptive femoral-femoral cannulation for cardiopulmonary bypass as well as the use of autologous blood transfusion using a cell-saver were matters that were discussed and instituted during this surgery. To the best of our knowledge, a rare gigantic thoracic ganglioneuroma requiring this multimodal approach has not been previously reported.

## Conclusion

The outcome of this complicated case was dependent on numerous factors that needed to be addressed perioperatively. In this endeavor, we would like to highlight the importance of an MDT approach as the main key factor attributing to its success. Meticulous preoperative planning and anatomical understanding, as well as taking pre-emptive measures to overcome any potential complications are indeed of paramount importance.

## Data Availability

Due to privacy and confidentiality concerns related to patient medical records and information, we are unable to provide direct access to the raw patient data. However, we are open to addressing specific data-related inquiries or requests on a case-by-case basis. Researchers interested in obtaining specific data points or seeking clarification on our findings are encouraged to contact the corresponding author.
